# Assessment of inpatient diabetes education throughout a structured questionnaire

**DOI:** 10.1186/1758-5996-7-S1-A173

**Published:** 2015-11-11

**Authors:** Thaís Lins Dos Santos, Flavia Fernanda Franco, Thalita Barreira Modena Cardim, Magda Tiemi Yamamoto, Rodrigo Bomeny de Paulo, Gustavo Daher, Rogério Silicani Ribeiro, Jose Antonio Maluf de Carvalho, Claudia Regina Laselva

**Affiliations:** 1 Hospital Israelita Albert Einstein, São Paulo, Brazil

## Background

Hospital admission is an opportunity for diabetes education. However, the efficiency of education may be compromised by stress, disease acuteness and the medication effects.

## Objective

To test the efficiency of patient education using a question form based on a tool developed by American Association of Diabetes Educators.

## Materials and methods

From January to May 2015, 92 patients were educated during hospitalization and were included in this pilot study. Since inclusion is ongoing, here we present partial Results. On average, age was 63+17 yrs. and time of diagnosis was 13+2 yrs. and 80 patients were on insulin. The average number of sessions were 2,8 (range 1-8). Previous A1c were available for 62 patients (average: 8,1%) and were above 7% in 46 cases. Patients were evaluated using questionnaire concerning the perceived importance of diabetes care and the knowledge about DM, nutrition, physical activity, glycemic control and medications. For those on insulin, there were additional questions concerning insulin administration and storage, glucose monitoring, response to hypo and hyperglycemia and foot care. For each question, patients scored from 0 (no knowledge) to 10 (full knowledge). After intervention, 64 (66%) were reevaluated with the same questionnaire. The major reasons for no reevaluation were discharge during weekend and discharge without notification.

## Results

**Figure 1:**
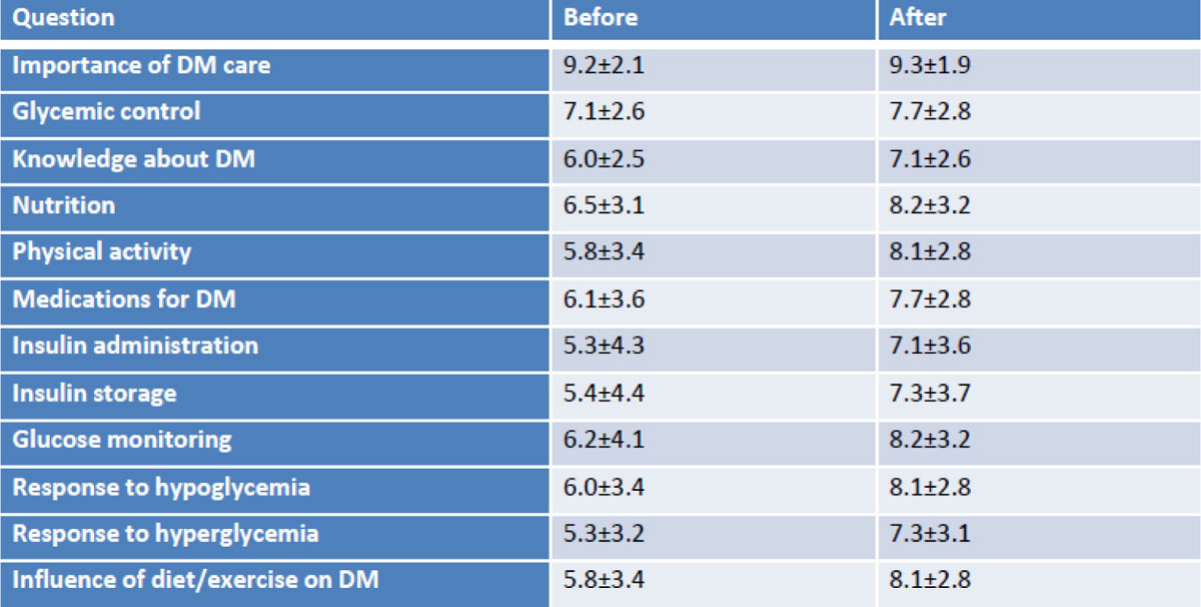
Scores for patients evaluated before and after education.

At baseline (n=92), patients highly scored the importance diabetes care (average score: 9.2±2) and glycemic control (7.8±3) but recognized a lower level of knowledge about DM (6.2±3), nutrition (6.8±3), physical activity (6.3±3) and medications (6.4±4). For those 80 on insulin, there lower scores for knowledge about insulin administration (6.2±4) and storage (5.7±4), glucose monitoring (6.6±4), response to hypoglycemia (6.5±3) and hyperglycemia (5.9±3). For patients evaluated before and after education, scores were described below (Figure 1).

## Conclusion

By performing a structured evaluation, we documented an improvement in diabetes knowledge of hospitalized patients. Besides every patient has some knowledge of the disease, after the education guided by the question form, all patients increase their grades for each subject.

